# Perennial biomass cropping and use: Shaping the policy ecosystem in European countries

**DOI:** 10.1111/gcbb.13038

**Published:** 2023-03-13

**Authors:** John Clifton‐Brown, Astley Hastings, Moritz von Cossel, Donal Murphy‐Bokern, Jon McCalmont, Jeanette Whitaker, Efi Alexopoulou, Stefano Amaducci, Larisa Andronic, Christopher Ashman, Danny Awty‐Carroll, Rakesh Bhatia, Lutz Breuer, Salvatore Cosentino, William Cracroft‐Eley, Iain Donnison, Berien Elbersen, Andrea Ferrarini, Judith Ford, Jörg Greef, Julie Ingram, Iris Lewandowski, Elena Magenau, Michal Mos, Martin Petrick, Marta Pogrzeba, Paul Robson, Rebecca L. Rowe, Anatolii Sandu, Kai‐Uwe Schwarz, Danilo Scordia, Jonathan Scurlock, Anita Shepherd, Judith Thornton, Luisa M. Trindade, Sylvia Vetter, Moritz Wagner, Pei‐Chen Wu, Toshihiko Yamada, Andreas Kiesel

**Affiliations:** ^1^ Institute of Biological, Environmental and Rural Sciences Aberystwyth University Aberystwyth UK; ^2^ Department of Agronomy and Plant Breeding I, Research Centre for Biosystems, Land Use and Nutrition (iFZ) Justus Liebig University Gießen Germany; ^3^ Institute of Biological and Environmental Sciences, School of Biological Sciences University of Aberdeen Aberdeen UK; ^4^ Department of Biobased Resources in the Bioeconomy (340b), Institute of Crop Science University of Hohenheim Stuttgart Germany; ^5^ Kroge‐Ehrendorf Lohne Germany; ^6^ UK Centre for Ecology and Hydrology Lancaster Environment Centre Lancaster UK; ^7^ Center for Renewable Energy Sources and Saving (CRES) Pikermi Attikis Greece; ^8^ Department of Sustainable Crop Production Università Cattolica del Sacro Cuore Piacenza Italy; ^9^ Institute of Genetics and Plant Physiology of the Academy of Sciences of Moldova Chisinau Republic of Moldova; ^10^ Institute for Landscape Ecology and Resources Management (ILR), Research Centre for Biosystems, Land Use and Nutrition (iFZ) Justus Liebig University Giessen Giessen Germany; ^11^ Centre for International Development and Environmental Research (ZEU) Justus Liebig University Giessen Germany; ^12^ Department of Agriculture, Food and Environment (Di3A) University of Catania Catania Italy; ^13^ Terravesta Ltd, Unit 4, Riverside Court Lincoln UK; ^14^ Team Earth Informatics Wageningen Environmental Research Wageningen Netherlands; ^15^ School of Chemical and Process Engineering University of Leeds Leeds UK; ^16^ Institute for Crop and Soil Science, Federal Research Centre for Cultivated Plants Julius Kühn Institute Braunschweig Germany; ^17^ Countryside & Community Research Institute University of Gloucestershire Gloucestershire UK; ^18^ Energene Seeds Limited, AIEC Office Block, Gogerddan Aberystwyth University Aberystwyth UK; ^19^ Institute for Agricultural Policy and Market Research Justus Liebig University Giessen Giessen Germany; ^20^ Institute for Ecology of Industrial Areas Katowice Poland; ^21^ Dipartmento di Scienze Veterinarie University of Messina, Polo Universitario dell'Annunziata Messina Italy; ^22^ National Farmers' Union Stoneleigh Park UK; ^23^ Plant Breeding Wageningen University and Research Wageningen Netherlands; ^24^ Department of Applied Ecology Geisenheim University Geisenheim Germany; ^25^ Field Science Center for Northern Biosphere Hokkaido University Hokkaido Japan

**Keywords:** BECCS, bioeconomy value chains, biomass utilisation, circular economy, energy security, farm subsidies, food security, integration into farm business, land availability, policy recommendation

## Abstract

Demand for sustainably produced biomass is expected to increase with the need to provide renewable commodities, improve resource security and reduce greenhouse gas emissions in line with COP26 commitments. Studies have demonstrated additional environmental benefits of using perennial biomass crops (PBCs), when produced appropriately, as a feedstock for the growing bioeconomy, including utilisation for bioenergy (with or without carbon capture and storage). PBCs can potentially contribute to Common Agricultural Policy (CAP) (2023–27) objectives provided they are carefully integrated into farming systems and landscapes. Despite significant research and development (R&D) investment over decades in herbaceous and coppiced woody PBCs, deployment has largely stagnated due to social, economic and policy uncertainties. This paper identifies the challenges in creating policies that are acceptable to all actors. Development will need to be informed by measurement, reporting and verification (MRV) of greenhouse gas emissions reductions and other environmental, economic and social metrics. It discusses interlinked issues that must be considered in the expansion of PBC production: (i) available land; (ii) yield potential; (iii) integration into farming systems; (iv) R&D requirements; (v) utilisation options; and (vi) market systems and the socio‐economic environment. It makes policy recommendations that would enable greater PBC deployment: (1) incentivise farmers and land managers through specific policy measures, including carbon pricing, to allocate their less productive and less profitable land for uses which deliver demonstrable greenhouse gas reductions; (2) enable greenhouse gas mitigation markets to develop and offer secure contracts for commercial developers of verifiable low‐carbon bioenergy and bioproducts; (3) support innovation in biomass utilisation value chains; and (4) continue long‐term, strategic R&D and education for positive environmental, economic and social sustainability impacts.

## Abstract 2

Perennial biomass crops (PBCs) can potentially contribute to Common Agricultural Policy (2023–27) objectives provided they are carefully integrated into farming systems and landscapes. Despite significant research and development (R&D) investment over decades in herbaceous and coppiced woody PBCs, deployment has largely stagnated due to social, economic and policy uncertainties. This paper identifies the challenges in creating policies that are acceptable to all actors and discusses the interlinked issues: (i) available land; (ii) yield potential; (iii) integration into farming systems; (iv) R&D requirements; (v) utilisation options; and (vi) market systems and the socio‐economic environment.

## 1 INTRODUCTION

Each successive Intergovernmental Panel on Climate Change (IPCC) Assessment Report (AR), now AR6 in 2022, adds more evidence of anthropogenic attributable climate change (https://www.ipcc.ch/reports/). Public awareness and responsive participation are rising through the actions of grassroots climate activists as well as high profile, globally recognised figures such as Greta Thunberg (Boulianne et al., 2020; Jung et al., 2020) and David Attenborough (Bonner, 2020; Burgess & Unwin, 1984). Almost all recently elected politicians in OECD countries have climate change mitigation as a top priority in their manifestos. Climate negotiations at the UN Conference of Parties in Paris in 2015 (COP25) and in Glasgow in 2021 (COP26) have ratcheted up emission reduction commitments through nationally determined contributions. Analysis by international agencies (e.g. International Energy Agency, Intergovernmental Panel on Climate Change) and national bodies (e.g. the UK's Energy Technologies Institute (2007–2018, www.eti.co.uk) and Climate Change Committee) show a significant role for biomass in negative/zero/low emission technologies, especially when biomass energy is combined with carbon capture and storage (BECCS) (Albanito et al., 2019; Bellamy et al., 2021; Donnison et al., 2020; Shepherd et al., 2021). In contrast, the European Union's (EU) Bioeconomy Strategy 2012 (European Commission, 2012) considered ‘biomass too good to burn’ because the supply of biomass is constrained. It emphasised using biomass in manufacturing bio‐based products, replacing fossil fuel‐intensive materials, including in the chemical sector, which requires fossil fuels for almost all products that are hydrocarbon based (Bugge et al., 2016; Fritsche et al., 2020).

Germany and the United Kingdom (UK) exemplify contrasting approaches. Both countries have a long tradition of forestry with 11.0 Mha (32% land cover) and 3.2 Mha (13% land cover) respectively. The UK has aspirations to increase forested areas to 15% of land cover. But, this paper focuses on dedicated biomass crops because the harvestable yield potentials are two to three times higher than from forestry. For agricultural biomass Germany has historically focussed on biomass from first‐generation annual crops, especially maize used in biogas plants (Fachagentur Nachwachsende Rohstoffe, 2021) whereas the UK has 10,000 hectares of perennial biomass crops (PBCs) mainly *Miscanthus* and willow, with small‐scale trials of short rotation coppice (SRC) and short rotation forestry (SRF) of species such as *Populus* (Defra, 2021b). However, in both countries, the liquid biofuels industry buys food crops (oil, starch and sugar) as feedstock and ‘food versus fuel’ conflicts are increasingly discussed (Muscat et al., 2022; Valentine et al., 2012).

**FIGURE 1:**
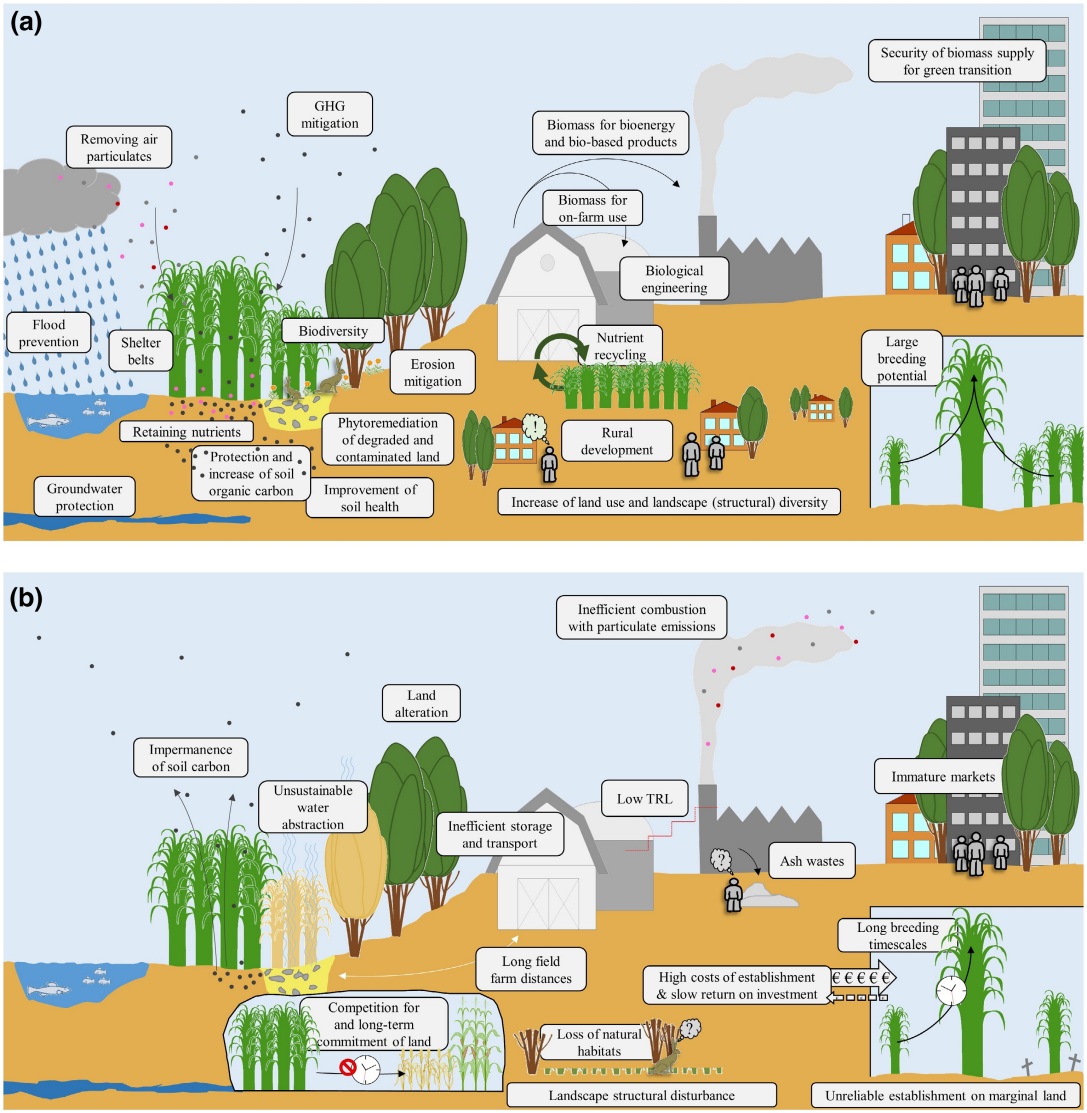
Perceived benefits (a) and potential dis‐benefits (b) associated with perennial biomass crops (PBCs) drawn from project results, the literature and practical experience depending on previous land use and social context. The term ‘Biodiversity’ refers to modifying landscapes providing habitats with lower disturbance than arable systems which have been shown to support birds, plants and small mammals especially on the transition zones (edges) between PBCs and the surrounding land use (Donnison et al., 2021; Lask et al., 2020). ‘Security’ refers to security of supply of biomass for the green transition and transformation of society.

Figure 1 shows the perceived benefits and potential dis‐benefits associated with PBCs drawn from project results, scientific literature and practical experience. Most of the terms in Figure 1 are self‐explanatory, but those we feel need clarification are explained in the legend. Considerable research effort over the last 30 years has resulted in a greater understanding of these benefits and dis‐benefits providing knowledge to help avoid negative consequences (Dondini et al., 2016; Martani et al., 2022; McCalmont, Hastings, et al., 2017; Milner et al., 2016). The cultivation and utilisation of perennial biomass causes significantly lower environmental impacts than annual crops (Kiesel et al., 2017) while providing a wider range of ecosystem services (Abreu et al., 2022; Von Cossel, Winkler, et al., 2020). However, as with all crops, PBCs require land, water and nutrients. They fall well within the land–water–food–energy nexus (Valentine et al., 2012; Vera et al., 2022). Introducing their production into an already established landscape requires some level of land‐use change with associated costs as well as benefits. PBCs are sometimes considered controversial because their production and use can be a carbon source or sink depending on climate, production conditions and practices, and especially the fate of fixed carbon in their use (Abreu et al., 2022; Pogson et al., 2016; Richards et al., 2017; Whitaker et al., 2018). A considerable number of long‐term land‐use change studies report on the carbon impacts of *conversion*, for example, from arable to PBCs and from grassland to PBCs (Dondini et al., 2016). The number of reported *reversion* studies from PBCs back to arable or grassland are increasing (Martani et al., 2022).

PBC research programmes over the past three decades have been driven by the need to reduce the use of fossil fuels in energy and material production and to maximise the environmental performance of growing raw materials for these purposes. Energy security per se had been a secondary objective until very recently but is now becoming much more prominent due to the fossil fuel and food security implications of the Russian invasion of Ukraine in early 2022. Up to the year 2021, total planted areas of PBCs had stagnated despite industrial partnerships embedded in public–private funded projects promoted by organisations such as the EU's Biomass‐Based Industries Consortium (https://www.bbi.europa.eu/). This stagnation may be attributed to several uncertainties, both for potential growers and supply chain managers; some related to technical aspects of crop management (Winkler et al., 2020) and others due to insufficiently joined‐up policy support from governments to create a sustainable market for the biomass produced (Bates et al., 2020). Multi‐actor communication is still lacking and scientists working on PBCs are increasingly being encouraged to engage with the public and policymakers through initiatives such as the EU's Common Dissemination Booster (CBD, www.cdbservices.eu) that aims to train researchers to communicate more effectively.

Figure 1 describes the perceived benefits and potential dis‐benefits associated with upscaling PBC deployment. However, the quality of evidence for the factors depicted varies dependent on the plant species and location, with limited evidence available across the whole life cycle of these long‐lived perennial crops. For example, the production of PBCs can have a positive or negative impact on soil carbon due to the complex relationship between initial soil carbon inherited from the previous land use and the organic material input from the subsequent PBC crop. Each land cover type has an equilibrium soil carbon; generally intensively managed areas of annual crops result in reduced soil carbon stocks while stocks under long‐term grassland, woodland and PBC crops are typically greater (Dondini et al., 2009, 2016; McCalmont, Hastings, et al., 2017; Pogson et al., 2016; Richards et al., 2017).

This paper, which is part of the special issue on ‘Valorization of Marginal Agricultural Land in the Bioeconomy’, arose from discussions between the coordinators of two EU projects: GRACE (GRowing Advanced industrial Crops on marginal lands for biorefineries, GA ID 745012) and MAGIC (MArginal lands for Growing Industrial Crops, GA ID 727698). The GRACE project established 100 ha of *Miscanthus* crop trials in 20 locations across Europe to advance and test technology readiness levels (TRLs) for novel hybrids from planting through to harvest for commercial upscaling of *Miscanthus*. The MAGIC project evaluated a wide suite of industrial biomass and oil crops for production on marginal land. Both projects have contributed knowledge to the benefits and challenges identified in Figure 1 but, like many other projects, were limited to 5 years, which is only 20%–30% of the possible productive lifespan of a *Miscanthus* plantation. This duration is insufficient to capture the full life cycle of a plantation; however, these projects do not stand alone as they add to a growing body of knowledge from three decades of research. The contributors believe the risk of inaction (i.e. no upscaling of PBCs, business as usual) to the climate to be greater than the risk of large‐scale deployment of PBCs for biomass production. We consider PBCs to be part of our ‘best bet’ solutions and therefore consider recommending their development to be adhering to the ‘precautionary principle’ used by the IPCC, which advocates ‘using a substantial body of evidence and experience to advance decision‐making rather than using a lack of evidence to excuse inaction’ (UN, 1992).

**FIGURE 2:**
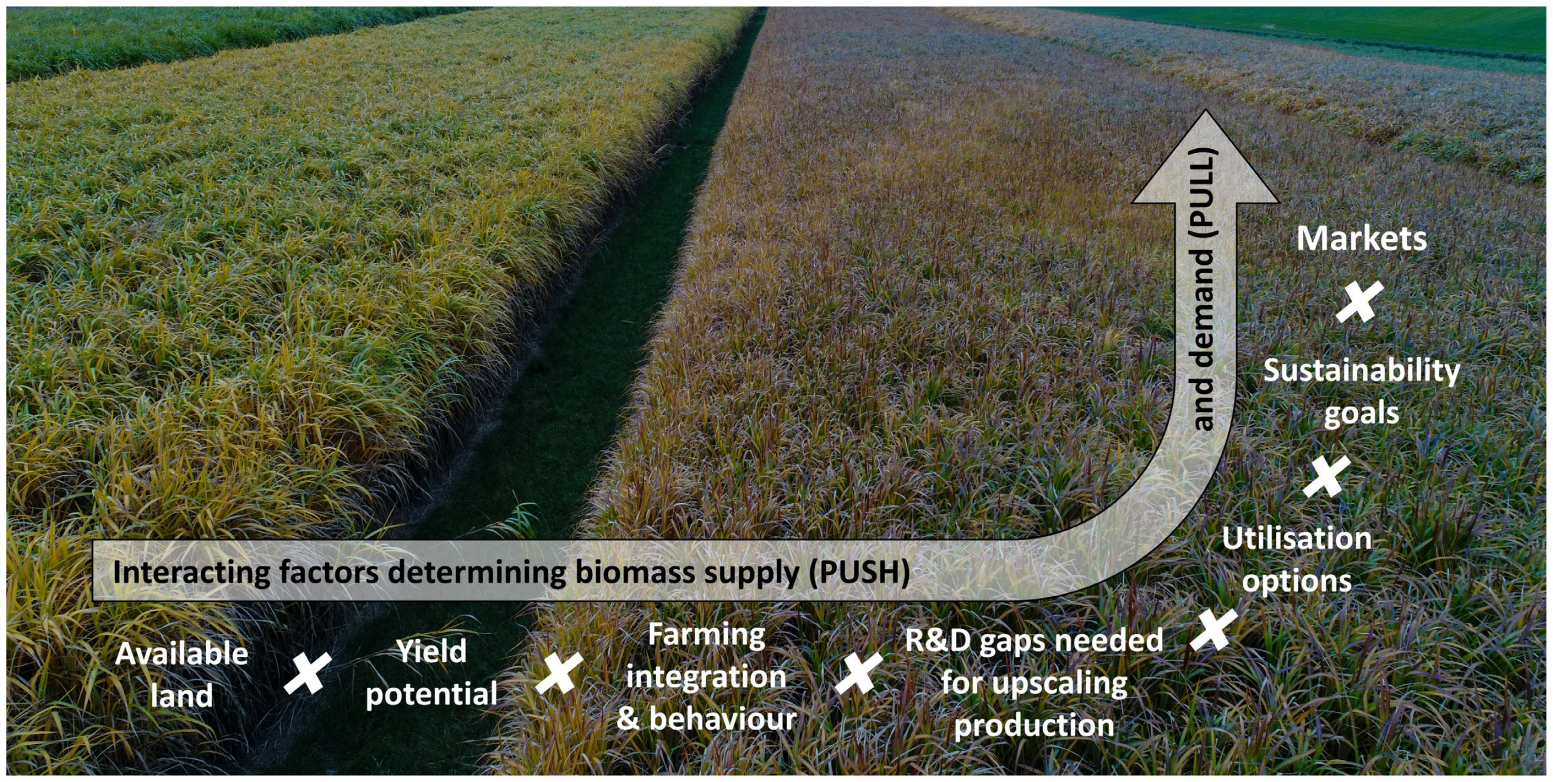
Factors involved in production PUSH and market PULL for PBC upscaling (discussed in sections below). These factors interact to determine the deployment opportunities for PBCs (production and utilisation chains) and identify broad areas for discussion on policy interventions.

To provide a structure for this review, we use a ‘pipeline model’ (Figure 2) divided into production push and utilisation pull. We use this structure to identify where barriers to upscaling exist, and their consequences from a production and utilisation perspective. This understanding of the impacts and interactions of different barriers then allows us to identify enablers required to tackle these barriers across the whole supply chain for different PBCs. The sequence for the *production side* is broken down into four ‘push factors’: (1) identifying available land; (2) yield potential; (3) farming integration; and (4) R&D gaps for upscaling production. On the *demand side* we consider three factors determining the market ‘pull’ (1) utilisation options, (2) impacts on sustainable development goals and (3) market drivers. We use this structure to identify where barriers to upscaling exist, and their consequences, from a production and utilisation perspective. This understanding of the impacts and interactions of different barriers then allows us to identify enablers required to tackle these barriers across the whole supply chain for different PBCs. Only if all actors in the pipeline can make a profit comparable to other potential activities with the same resources (i.e. benefits outweighing opportunity costs) will upscaling occur.

## 2 PUSH FACTOR

### 2.1 Available land

How much land could be used for PBCs without detrimentally affecting essential food production or ecosystem services? This is a complex question to answer, as there are many interacting variables including population demographics and distribution, diet, technological advances and political shocks (Lewandowski, 2015; Von Cossel, Wagner, et al., 2019). One suggestion is the concept of land sparing associated with sustainable intensification (Godfray & Garnett, 2014; Lamb et al., 2016). This was tested by the EU Common Agricultural Policy (CAP) ‘set‐aside’ policies of the early ‘90s where it was found that leaving 10% of arable land fallow failed to reduce overall food production in the EU as predicted by Hodge (2007). Later policies allowed the planting of crops for industrial purposes on ‘set‐aside’ land, but from 2007, adverse weather reduced yields and ‘set‐aside’ was discontinued. ‘Set‐aside’ was mandated as 10% of arable land on each farm and did not discriminate between highly productive and less productive land. The scope for allocating less productive land to set‐aside was limited because farmers could not trade the set‐aside commitment to land which was marginal for economically viable food production. Analysis in the H2020 (sustainable exploitation of biomass for bioenergy from marginal lands) and MAGIC projects calculated a land resource of 95 and 69 million ha respectively of marginal agricultural land in Europe that could potentially be used for PBCs (Elbersen et al., 2018; Gerwin et al., 2018). Thus, the spatial configuration of historical set‐aside land is unlikely to match the requirements for optimal deployment for PBCs. A GIS analysis of suitable land for PBCs in the UK indicated that out of the total UK agricultural land (arable and improved or rotational grassland) area (18 million ha) 1.4 million ha (~8%) could be planted with PBCs without reducing food production capacity (Lovett et al., 2014; Smith et al., 2014), which is close to the 10% mandated in EU set‐aside policy. In Brazil, the US, EU and UK, a large amount of land is used to produce high input annual crops (food crops and silage maize) utilised for bioenergy (‘first‐generation’ bioenergy crops such as oil seed rape being used in bio‐diesel production or maize into bio‐ethanol and biogas). Concerns about indirect land‐use changes were triggered by policies supporting the use of first‐generation bioenergy crops, first in the US (Searchinger et al., 2008) and then in the EU by the Institute for European Environmental Policy (IEEP) (Kretschmer & Baldock, 2013) due to a failure to meet sustainability criteria. Focussing PBC planting on land less suitable for food production (often referred to as marginal agricultural land) has been proposed as an effective way to mitigate indirect land‐use change risks (Traverso et al., 2021) and improve habitat for biodiversity. Marginal land categorisation is complex and comprises many factors: soil texture (Gerwin et al., 2018), aspect ratio, drainage, climate, stoniness, altitude etc. (e.g. MAFF, 1988), and must also consider sociological and economic contexts (Helliwell, 2018; Shortall, 2013). Recent programmes, including the EU MAGIC project (https://magic‐h2020.eu/), have worked to improve these definitions and quantify potential land area to better evaluate the impact of land‐use change to PBC production (Elbersen et al., 2018; European Commission et al., 2022; Von Cossel, Lewandowski, et al., 2019). At a European level, spatial analyses are beginning to use remote sensing to identify abandoned, degraded or contaminated lands that could move from annual to perennial crop production, that is, available for PBCs, afforestation or rewilding (European Commission et al., 2022; Meijninger et al., 2022).

It has been argued that a reduction in consumption of livestock products is required to ‘free up’ land for biomass feedstock cultivation as well as for afforestation and restoration of other natural ecosystems (CCC, 2020b). Although livestock products are an important component of the diet of the majority of people in developed economies, it may be desirable to moderate their intake on the grounds of health as well as the multiple environmental impacts of their production (Willett et al., 2019). Recommended dietary changes range from a modest 20% cut to a halving of per capita consumption of red meat and dairy products (CCC, 2020b), with consequent reductions in livestock numbers of a third or even a half (Scheffler et al., 2021). A recent analysis found that 75% of agricultural land use in Germany is used for livestock production (mainly grain fed pigs) (Scheffler et al., 2021) while globally around 40% of all arable crops grown are used to feed livestock for meat, egg and dairy production (Mottet et al., 2017).

However, farmers’ organisations point out that, in the UK and other European countries, much of the pasture land used for cattle and sheep grazing is incapable of supporting food crop production, and that cutting livestock numbers risks displacing production to farming systems overseas with a higher carbon footprint (The Facts about British Red Meat and Milk: https://www.nfuonline.com/updates‐and‐information/rethinking‐ruminants‐member‐toolkit). The National Farmers Union of England and Wales anticipates that changes in food consumption and production are more likely to be gradual than rapid and advocates technological and systemic advances (such as feed additives, novel feed proteins and breeding) to drive productivity growth and reduce greenhouse gas (GHG) emissions from livestock production (Scurlock, pers. comm.). If landowners are to be persuaded to convert some land from livestock to PBCs, the income to producers from growing PBCs needs to be at least as economically attractive as livestock farming. This should consider that many small‐scale farmers depend on subsidies and social welfare payments to survive. Comprehensive policy support may be necessary to allow farmers and supply chains the confidence to transition to these new low‐carbon enterprises, and is likely to be fundamental to achieving the net zero ambitions of the agricultural sector (CCC, 2020c; Reay, 2020).

In addition to the challenges of identifying potentially available land, there are also important considerations of how PBCs can be spatially integrated into the landscape to maximise co‐benefits for the ecosystem while minimising negative environmental impacts such as nutrient emissions (Dauber & Miyake, 2016; Tscharntke et al., 2005; Von Cossel, Winkler, et al., 2020). PBC strip/alley plantings into ‘readily harvestable‐hedgerows’ could provide shelter, erosion control and landscape connectivity supporting wildlife and biodiversity (Dockerty et al., 2012; Kraft et al., 2021; Lamerre et al., 2015; Tsonkova et al., 2012). Implementation details would depend on the specific site attributes, such as soil texture, rainfall, current land use and landscape type (Tscharntke et al., 2005). To implement these would likely require a ‘farm level’ environmental impact assessment and a system for Monitoring, Reporting and Verification (MRV) certification with payments for ecosystem services using multi‐dimensional metrics (Milner et al., 2016; Von Cossel, Wagner, et al., 2020).

The developers of new land‐use policies to support farmers in the UK and EU are grappling with this due to a scarcity of robust long‐term quantitative evidence about land‐use transition to PBCs from alternative land use. Expert judgments by environmental scientists will be key to the successful implementation of such nuanced landscape policies. In the UK, Department for Environment, Food & Rural Affairs (Defra) developed a 25‐year environment plan (Defra, 2018). This largely retains the 10 objectives of CAP (European Commission, 2022) which will be upheld in the post‐Brexit English Environmental Land Management Schemes (ELMS, (Defra, 2021a)) and Welsh Sustainable Management Scheme (Welsh Government, 2022). Members of the PBC community have provided input to recent consultations on these new post‐Brexit schemes. To date, however, these new schemes are still being developed.

As scientists, we would hope that data at the whole European level, from trials combined with crop modelling and remote sensing in Geographic Information Systems (GIS), would be used in planning and supporting spatially explicit land use, including PBC plantings.

In conclusion, these debates on land availability are nuanced, but extensive integrated assessment modelling, both within‐country and EU‐wide, does support the expansion of PBCs (CCC, 2020a). Based on these modelling outcomes and our experience we conclude that, with appropriate ongoing MRV schemes, a simple EU‐wide target of 10% of total existing agricultural land (arable and rotational grassland) for PBCs is large enough to deliver sufficient feedstock to develop the sustainable biomass‐based industries required, but small enough to provide protection for current food production capacity, water resources, biodiversity and the environment. *We recommend therefore that agricultural and related policy support PBC production to 10% of the agricultural area and for this to be included within the following CAP period from 2028 to 2032*. Clearly, however, pursuit of such a target must also consider the local context and conditions, such as changing climatic ranges for crop suitability, outbreaks of pests, diseases and political events (such as the Russian invasion of the Ukraine (Ben Hassen & El Bilali, 2022; Bentley, 2022; BÖR, 2022; Esfandabadi et al., 2022; Glauber & Laborde, 2022)).

### 2.2 Yield and carbon capture potential

To optimise the economic return from PBCs and the carbon savings or GHG removals delivered by these crops, we need to maximise above‐ground yield and below‐ground soil C sequestration while minimising field‐based GHG emissions. Over 200 field trials have been planted across Europe to study the establishment, production potential and environmental costs and benefits of PBCs. We estimate that the number of trials performed on perennial rhizomatous grasses are *Miscanthus* spp. (~100), *Panicum* spp. (~20) and *Phalaris* spp. (~20). For short rotation coppice (e.g. *Salix* spp.*, Populus* spp.) there are probably ~100 trials but the yield series from the bi‐ or triennial harvests are typically reported for only two cycles (due to typical 3–5‐year research funding), with longer time series rarely reported in the literature. Yield series from short rotation forestry (SRF, e.g. *Populus* spp., *Robinia* spp., *Eucalyptus* spp., *Paulownia* spp. and *Alnus* spp.) are even more scarce due to the 5–20‐year typical rotation length and the smaller land areas devoted to SRF.

**FIGURE 3:**
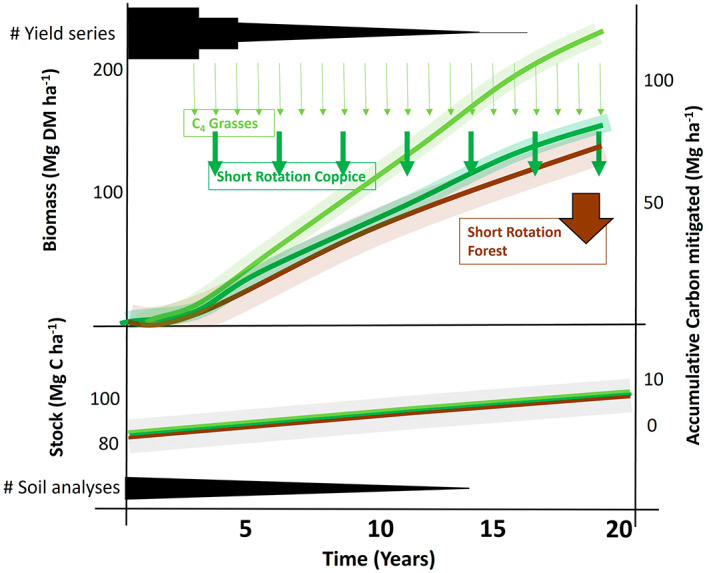
Top panel: Above‐ground accumulated harvest yields for three different PBC systems with different harvest cycles: annually – (C_4_ grasses e.g. *Miscanthus*/ Switchgrass), every 2–4 years (short rotation coppice e.g. Willow), every 5–15 years (short rotation forestry e.g. Poplar) indicated by the coloured coded arrows (top, redrawn from Hastings et al., 2012). The bottom panel shows the accumulative carbon stock for soil carbon for the 0–30 cm as informed by Dondini et al. (2009). ‘Sankey style’ black line thicknesses schematically indicate how the numbers (#) of measured yields (see Table S2 for details) and soil carbon experiments diminish well before the expected crop lifespans are reached.

The yields of PBC's and SRF vary dependent on the harvest cycle/rotation length and time since planting, with the fastest growth rates in the early years in perennial grasses and woody crops (see Figure 3). To project above‐ground harvestable yields, studies have used traditional crop models with parameters adapted to PBCs using field trial data (MiscanFor (Hastings, Clifton‐Brown, Wattenbach, Mitchell, & Smith, 2009), PopFor (Henner et al., 2020)), Switchgrass (Di Vittorio & Miller, 2014; Liu et al., 2022). Assumptions and generalisations in these models are under constant review, as new datasets for climate, soil and crop growth become available. Generalising model growth parameters to upscale the yield projections on maps are performed using rasterised climate and soil data (Hastings et al., 2014; Hastings, Clifton‐Brown, Wattenbach, Mitchell, Stampfl, & Smith, 2009; Shepherd, Littleton, et al., 2020). Yield potentials estimated by these crop production models are then used to determine potential soil carbon changes and GHG emissions from these crops (Pogson et al., 2016; Richards et al., 2017).

Output maps of potential yield are dependent on the spatial resolution of input climate, land use and soil property maps, which are often at 1 km resolution. In addition, the temporal nature of climate and weather data are not well reflected by the daily and monthly averages used in these datasets. They smooth over extreme events in particular. Predicted yields tend to be the average of several years using the dominant soil type in each spatial grid cell. While this is good enough for planning of large‐scale use of biomass, this is not spatially and temporally explicit enough to predict yields at field levels (Shepherd et al., 2021). This would require knowledge of soil types across the field, depth of the water table and local microclimate drivers such as slope and aspect affecting radiation and exposure to wind. Both these impact on water balances and air mixing (inversions and stratifications) which cause temperature extremes, for example, frost (frequency and severity) and heat and moisture stress. These factors all control crop growth and development within the growing season. Differences between modelled yield potentials and farmer measurements can be explained by variation in crop establishment rate (Shepherd, Clifton‐Brown, et al., 2020), effectiveness of crop management, and by missing fractions in commercial harvesting (e.g. stubble residue heights (Magenau et al., 2021)).

Maximising above‐ground yield is a significant factor in the carbon savings delivered from PBCs. However, changes in soil carbon stocks and other GHGs which need to be accounted for in overall carbon budgets, or equivalent (see McCalmont et al., 2018), as land‐use change to, and reversion from PBCs can result net emissions (Ouattara et al., 2021; Rowe et al., 2020). In the UK, the ELUM project showed that converting annually cropped land to PBCs and SRF typically resulted in a soil carbon gain, but planting on rotational grassland gave more variable results. This was a large UK‐wide study, which provided new data to be used in modelling, but there are still significant uncertainties over the longer‐term trends reported due to a lack of empirical, time‐series data. There are very few trials where soil carbon is measured properly with adjustment for changes bulk density over time, sampling before planting or with an appropriate paired site, appropriate depth of sampling (min of 30 cm) and with sampling conducted after a sufficient length of time to detect statistically meaningful stock changes (Kravchenko & Robertson, 2011; Rowe et al., 2016). The delta 13C shifts associated with converting to PBCs with C_4_ photosynthesis help with detection of new carbon such as in Dondini et al. (2009). In addition, actual GHG flux measurements in paired sites representing land‐use change and original land use are only just starting to be undertaken, one example being the PBC4GGR project in the UK (https://pbc4ggr.org.uk), where verification and reporting of soil carbon change will be carried out by combining on‐site eddy covariance monitoring with modelling (Dondini et al., 2016; McCalmont, McNamara, et al., 2017). At the end of the crop lifespan the root and rhizome biomass of the PBCs is incorporated in the soil by maceration and is decomposed relatively quickly (Martani et al., 2023). It should be noted that reversion to previous land use will likely result in a return over time to soil carbon levels commensurate with that land use and therefore should not be used for carbon credits.

*We recommend policy makers put in place measures to ensure that the performance of these new crops is monitored by measuring yields on farms over the crop lifespan* (*~15 years*) *to create a best practice knowledge base. This could be a requirement for receiving any financial incentive related to cropping PBCs*.

### 2.3 Integration into farm businesses

Cross et al. (2021), following an analysis of the effectiveness of bioenergy policy in UK and Nordic countries, argues that each country has a unique landscape of environmental, regulatory and energy factors that mean that it is hard to extend the lessons learned from bioenergy policy implementations from one country to another. There is a need for holistic policy support inclusive of all land uses (Rowe et al., 2022).

As with any new cropping system, innovation or policy instrument, many factors interact and affect land managers' decisions on whether to grow PBCs. There is a growing body of work in the UK that identifies social, economic, technical and political barriers to integrating PBCs into farming systems as well as identifying ‘enablers’ to facilitate change. At the farm(er) level, identity, values and culture have been shown to determine perceptions of marginal land and the symbolic value of food production, while unfamiliar agronomy and compliance requirements attached to economic incentives have negatively affected attitudes towards growing PBCs (Helliwell, 2018; Shortall, 2013). Economically, the advantages in terms of diversifying farmer income streams and in providing a low maintenance crop suiting certain farm system workloads is recognised. However, there has been resistance to adoption due to attitude and perceived risk of loss (Anand et al., 2019). Such factors include: upfront investment, long‐term commitment of land, potential crop failure, yield variability that is not protected by crop insurance, competition with alternative land use (including other non‐food options), immature markets, limited number of end‐users and lack of long‐term market certainty. For example, the perception of risk of bioenergy company failure increased following historic precedents (e.g. the pyrolysis plant ARBRE near Selby, UK (Barker, 1996; YorkPress, 2002)). To plant PBCs, growers need to have identified a market for the life of the crop (Rowe et al., 2022). The capacity to absorb these risks varies according to farming system characteristics (e.g. size, tenure, level of investment in other enterprises and a positive grower attitude towards innovation of new products and markets). In Europe, capacity for uptake of PBCs is greater at large arable farms (farms >100 ha account for 50% of the utilised agricultural area (Eurostat, 2016)) with appropriate infrastructure and machinery. In the UK, many tenant farmers and contract farmers have tenure agreements that are shorter than PBC crop lifespans which is a strong disincentive. Farmers have also been found to prefer the flexibility of annual crops which allow them to respond to changing commodity prices. Uncertainties associated with future policy instruments such as emerging carbon markets, as well as the food and energy security debates and volatile cereal and oil crop prices associated with the war in Ukraine, are all disincentives to commit to PBCs (Ingram et al., 2023). On a more positive note, evidence for the ecosystem service benefits of PBCs is building, for example, using PBC strips (which could include agro‐forestry) in open arable landscapes to promote biodiversity (Kraft et al., 2021). These aspects of PBCs are viewed positively by farmers and large‐scale land managers (such as the UK's Royal Society for Protection of Birds, the National Trust, the military, Crown Estates, golf course owners etc.) and could support PBC integration into future land management payments in the EU and UK focused on environmental and public goods, but this will require the development of novel measurement, reporting and verification methods.

Within farming systems, supply chain intermediaries or innovation brokers can be influential in increasing uptake (Helliwell et al., 2020). This is reflected in eastern Britain, northern France, southern Germany (Von Cossel, Amarysti, et al., 2020) and at several locations in eastern Europe, where pioneer biomass supply chain companies are operating. These companies provide expertise to growers and make connections to markets. Their business models vary regionally with either bioenergy or bioproducts, with some offering long‐term contracts related to crop lifespan with guaranteed indexed prices related to biomass quality. Some contracts smooth cash flow to overcome costs during plantation establishment years through financial support mechanisms. The importance of these companies in building confidence in the farming community and developing market and industrial capacity and lobbying government is clear. Contracts are being specifically developed to overcome the effects of inadequate markets (Adams & Lindegaard, 2016; Kärcher et al., 2015; Piterou et al., 2008).

For both growers and supply chain companies to expand production of PBCs, they need stronger, longer and more integrated policy support and the confidence that this support will remain consistent over time scales that are relevant to the economic performance of perennial crops. Small adjustments such as the relatively recent inclusion of PBCs in CAP ‘greening payments’ (Emmerling & Pude, 2017) have helped but are insufficient. In the UK, between 1998 and 2005, establishment grants for PBCs, in conjunction with markets created by Drax power, grew the areas of production from less than 1000 ha to over 10,000 ha for *Miscanthus* and over 3000 ha for willow. A similar increase was observed in Germany in cup plant (*Silphium perfoliatum* L.) cultivation for biogas feedstock production where the area under cultivation increased from 500 to 10,000 ha from 2016 to 2021 due to a cap on the proportion of maize used in biogas production (‘Maisdeckel’) in the revised EEG (EEG, 2021) and the development of scalable direct sowing agronomies (Cumplido‐Marin et al., 2020). This increase is put forward as a success story, but it should really be contextualised with the UK 2012 Biomass Strategy target which set a target of 350,000 ha for PBCs (DECC, 2012). However, when compared with the increase in *Brassica napus* (oil seed rape) planting from essentially 0 to 400,000 ha in 10 years in the UK (from 1980 to 1990) and then onto 700,000 ha by 2013 (Defra., 2014) demonstrates the contrasting adoption rates between annual and perennial crops.

**FIGURE 4:**
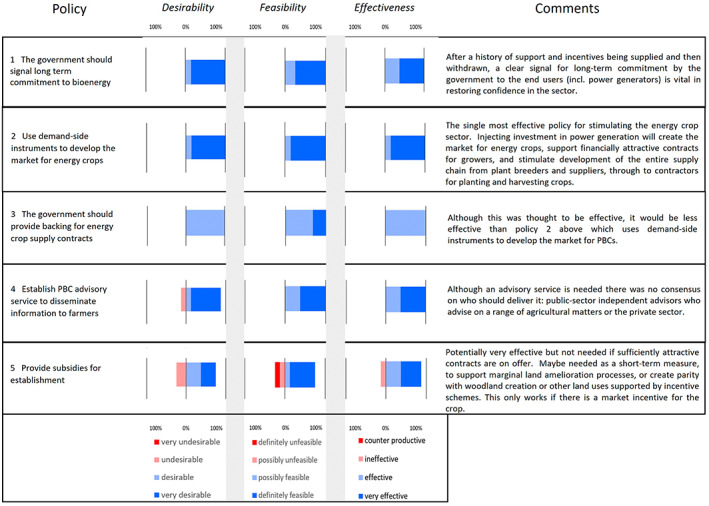
Five policies (out of a set of 13) ranked from top to bottom for promoting adoption of PBCs by UK farmers. Ratings of ‘desirability’, ‘feasibility’ and ‘effectiveness’ of the policies from a Delphi panel of nine experts.

Adams and Lindegaard (2016) identified similar obstacles in a policy review for the period 1990–2015. More recently, in a study in 2020 using a Delphi approach (Dalkey & Helmer, 1963), UK PBC stakeholders (from farmers, industry and academia) identified the top 5, out of 13, biomass policies according to categories for ‘desirability’, ‘feasibility’ and ‘effectiveness’ (Figure 4) (Ford, 2021). A summary of the discussions on each policy is shown in the comments.

The panel recognised the need for long‐term commitment and strongly recommended policy intervention at the end of the PBC supply chain to provide electricity generators with the financial security needed to offer attractive contracts to farmers, which would then in turn stimulate development of the full supply chain (Figure 4). But, as mentioned earlier, incentivising large‐scale end‐users may not pull through small‐scale supply chains. These comments are also reflected in other stakeholder workshops concerning feedstocks for negative emission technologies (Vaughan & Gough, 2016). This view was also reflected in a survey of 20 existing *Miscanthus* growers who identified the largest barrier to extending their *Miscanthus* crop areas was the establishment cost (von Hellfeld et al., 2022). The Delphi study stakeholders contributing to Figure 4 identified that further investment in breeding‐agronomy research to improve establishment speed and in the promotion of advisory support for growing PBCs was desirable. However, the balance of public and private investment was debated; some panellists supported including PBCs in the UK Environmental Land Management Schemes designed to deliver public goods, while others felt that the benefit of PBCs was not as great as other uses of land, such as woodland creation, and political opposition to inclusion could be expected. Although this Delphi study (Figure 4) involved only a small group of PBC experts in England these views are consistent with analysis in 2022 based on 20 semi‐structured interviews and two workshops with a range of *Miscanthus* and SRC willow growers in England (Ingram et al., in preparation). They are also supported by workshop analysis (74 stakeholders) where technical, social, political and economic barriers to the sustainable growth of the UK energy crop sector were identified (Rowe et al., 2022). Both studies emphasised the need for policy integration across government departments, government continuity and communicating strategic priorities which would help build market confidence. They also identified the need for clarity with respect to policy incentives such as the Environmental Land Management Schemes agri‐environmental schemes, planting grants and any emerging carbon markets.

These studies demonstrate that further social science research is urgently required, involving a wide variety of stakeholders, thus taking a multi‐actor approach. Social science offers several approaches for understanding how to upscale PBCs. These can relate to the farm(er) level, for example: a socio‐psychological and behavioural perspective (Mills et al., 2017; Warren et al., 2016); or understanding farmer adoption decision‐making processes as affected by factors such as individual and farm characteristics, market structure, social networks and media influence (Burli et al., 2021); or innovation adoption framing (Pannell et al., 2006), which considers trialability, compatibility, complexity, relative advantage, observability and ‘absorptive capacity’ for new practices and technologies. Alternatively, a socio‐technical systems perspective can be applied to recognise the various interdependencies between technological and social aspects (cultural, ethical) of any transitions; or an innovation systems approach (including Agricultural Innovation Systems) (Silveira & Johnson, 2016). These underpin the transition approach to understanding innovation diffusions widely applied to other agricultural contexts (see discussion below).

Societal acceptance is a further area where social science can contribute. As widespread PBC plantings will change the visual landscape, it is important to understand the perceptions of all stakeholders, including the public, of this change and its benefits and dis‐benefits. Although the visual impact of PBCs is reportedly less contentious than for other renewable energy systems, which are seen to threaten the ‘rural aesthetic’ (Dockerty et al., 2012; Ingram et al., 2022; Karp et al., 2009), views on landscape change may create barriers. Stakeholders have diverse value judgements on aspects of biodiversity and ecosystem services and attach different symbolic meaning to land (Eaton et al., 2019). This is particularly pertinent given current debates about food and energy security, and net zero. There is scope therefore to include deliberative techniques with communities to try to reduce or transform specific local economic impacts and enhance community and wider societal ‘buy‐in’. *We recommend that PBC development be community based with active involvement of local communities in project development with priority given to generating benefits for communities*.

### 2.4 R&D needed for upscaling production

Over the last 20 years, public‐supported R&D with industry involvement and coordination between national and EU‐funded projects has delivered significant advances in our ability to scale‐up PBC deployment. An analysis of the development steps (genetic resource collection and characterisation, breeding, propagation, agronomy, harvest, transport and storage, pre‐treatment and valorisation) from a selection of PBC projects involving *Miscanthus* over the past decade is shown in Appendix S1. This chronological analysis shows how successive projects have attempted to plug crucial knowledge gaps. In large projects, parallel development in different steps has been attempted to accelerate holistic system developments, making chains that connect production with utilisation.

Public sector investment to collect genetic resources following the guidelines on the Convention on Biological Diversity (Huang et al., 2019) has been necessary as the original material exists in diverse countries for all PBCs. For example, *Miscanthus* is indigenous in East Asia: China, Taiwan, Korea and Japan. Phenotypic characterisation of genetic resources in‐situ and in field trials across Europe has compared the yield potential and compositional quality of different accessions in different meteorological and physical environments (Clifton‐Brown et al., 2019). The breeding pipeline for new clones/ hybrids/varieties involves genotypic and phenotypic data management including complex traits such as flowering time and flowering synchronisation (Jensen et al., 2011, 2013) to make seed. A chain of field trials of different scales are used to identify and upscale the most promising hybrids to reach technology readiness levels (TRLs) 6–7, equivalent to successful prototype demonstration. Typical start to finish testing durations are 12–15 years for the grasses *Miscanthus* and switchgrass and 15 for willow and 22 years for poplar (Clifton‐Brown et al., 2019).

EU programmes have been effective in building up multi‐location trial networks for evaluation of new hybrids and how well they are matched to different environments (Kalinina et al., 2017; Kiesel et al., 2017; Nunn et al., 2017). Continuity of these trials over relevant timescales beyond the EU programmes depends on national funding arrangements which tend to be patchy, jeopardising progress.

Beyond plot trials, there is much to do in agronomy and crop management to upscale to commercial fields. For the UK the Climate Change Committee (CCC) has calculated, with checks and balances on other land‐use requirements, that PBC planting needs to be extended by 23,000 ha per year from 2020, reaching around 700,000 ha by 2050 to make their expected contribution to ‘Net‐zero carbon emissions target’ (CCC, 2020a). However, willow areas fell by 170 ha p.a. in the UK between 2015 and 2020, while *Miscanthus* increased by only 276 ha p.a. Indeed, upscaling to planting 23,000 ha per year of *Miscanthus* through rhizomes would require about 2000 ha of nursery fields on a 2‐year cycle with a similar area required for willow. While in‐vitro techniques have higher multiplication rates (~1000 in a year, depending propagation amenability (Kai Schwarz, pers. comm.)) they are three times more expensive (Xue et al., 2015). This has been the driver for developing *Miscanthus* propagation by seed which has multiplication rates from 2000 to 5000. This only requires between 10 and 20 ha of land in a southerly location where the parents flower to produce sufficient seed to achieve the UK CCC upscaling target. Large inter‐annual variations in weather present new challenges to crop establishment, such as early spring or summer droughts, or floods during planting periods (https://www.ipcc.ch/report/sixth‐assessment‐report‐cycle/). However, innovations in planting and agronomy such as mulch films are being developed to cover these challenges (Ashman et al., 2023).

For *Miscanthus*, harvests, storage and transport logistics and pre‐treatment options need further work after the GRACE project. In addition, spatial harvest yield monitoring in commercial plantings is required to inform these developments to optimise yield and minimise environmental impact. *We recommend policy makers put long‐term commitments to publicly supported R&D and coordination between national and EU‐funded projects needs to continue. Industry involvement in projects is essential to the commercial translation of the technologies developed*.

## 3 PULL FACTORS

### 3.1 Utilisation options

The fifth step identified in Figure 2 is where the biomass enters value chains. The push factors 1 to 4 in Figure 2 impact the potential quantity and quality, spatial and temporal availability of biomass, but without the end‐to‐end value chains this potential will not turn into reality. These currently are: (1) biomass for energy, (2) biomass for energy with carbon capture and storage, (3) biomass for chemicals and materials in bioproducts to replace high carbon alternatives and (4) biomass for livestock bedding and fodder. Cascaded uses for the different biomass fractions are being actively encouraged for the circular bioeconomy.

For bioenergy there are many national initiatives (Cross et al., 2021) but it has long been argued that a simpler ‘volume market’ is a better way to initiate sector growth. In the UK, favourable policies for bioenergy have supported simple straw‐burning power stations with a total installed capacity of ~160 MW in 2022 (www.eco2uk.com). These currently capture neither heat nor CO_2_ but, depending on transport distances, still only emit 21.3 kg CO_2_ MWh^−1^ which is an order of magnitude less than gas (Hastings et al., 2017); however, more could be done to maximise GHG mitigation. In addition to providing much‐needed renewable energy, these straw‐burning power stations have established domestic biomass supply chain actors and developed the expertise needed to deliver more ambitious plans for Bioenergy with Carbon Capture Storage (BECCS). Drax power station, the largest generator in the UK, is now the largest *biomass powered* station in the world and will completely stop burning coal in 2023. In 2019, Drax proved the industrial scalability of CCS with biomass flue gases and with its proximity to the North Sea oil fields can provide 500 years of geological storage for carbon captured (Hastings & Smith, 2020). Consequently, Drax and the UK government plan to build a full scale BECCS facility between 2024 and 2027. Currently Drax's biomass is largely imported from managed forests in Eastern North America (5 million tonnes p.a.). This supply chain was developed by pelletising wood unsuitable for timber products that were previously considered waste, thus creating a sustainable fuel source. Reuters poll on carbon price in 2021 indicated that the price must be increased to more than $100 (up to $250) per tonne to limit warming to 1.5°C (Bhat, 2021). At this level the carbon price will cover the costs of the CCS component (Hastings & Smith, 2020). As other countries expand biomass use, prices are expected to rise (Bates, 2017) with increasing importance on indigenous biomass production driven by global shocks such as the recent Russian invasion of Ukraine.

In Germany, it was energy policy rather than agricultural policy that led to the largest recent changes in agriculture. The EEG (Renewable energy law) supported the production of green electricity (Murphy‐Bokern, 2016). Feed‐in tariffs were granted to farmers or biogas plant operators for producing electricity from biogas. This policy intervention led to a boost of investments into biogas plants and today Germany has about 8600, mostly farm‐based, biogas plants (FNR, 2021b) using manure in combination with maize. As a result, silage maize production for biogas rapidly increased until 2011 and since then remained constant at approximately 2.65 million ha (FNR, 2021a). Due to a revision of the EEG in 2012, further expansion of biogas‐based electricity generation was largely stopped due to reduced guaranteed feed‐in tariffs for biogas electricity from energy crops. PBCs such as *Silphie* or *Miscanthus* have the potential to improve the environmental sustainability of the biogas substrate production (Kiesel et al., 2016), but many biogas plants are getting close to the end of their for 20‐year guaranteed feed‐in tariffs. To avoid decline in biogas production capacity there is a requirement to develop new policies to develop economically viable business models for the post‐EEG period and to shift their substrate mix towards residues and PBCs from marginal land.

In the EU, the vision for the Biomass‐Based Industries (BBI) initiative (2014–20) was ‘a competitive and sustainable Europe leading the transition towards a bioeconomy, while decoupling growth from resource depletion and environmental impact’. The BBI promoted products not energy, the cascaded use of biomass feedstocks and their use in long‐lived products such as building materials. This has been recently replaced by the Circular Bio‐based Europe Joint Undertaking (CBE) (2021–2031) (https://www.cbe.europa.eu). In addition to the cascaded uses, the CBE initiative is pushing for whole system circular thinking where the end of life for one product is the beginning of life for another bio‐based product. At farm level this could include traditional uses of biomass residues for livestock bedding, where soiled biomass becomes a feedstock for anaerobic digestion to produce bioenergy after which the digestate is turned into fertilisers which are used to grow more biomass. Ambitious projects will be needed to translate these simple concepts into commercial practice.

*We recommend that financial and policy support should be achieved by increasing carbon pricing which will encourage the development and use of low greenhouse gas emission energy and materials. This carbon pricing support should be designed in such a way that all actors in the supply chain, including farmers, reap the benefits*. Implementation could be accelerated by the current energy shortage caused by Russia's invasion of Ukraine.

### 3.2 Market systems, the socio‐economic environment and sustainability goals

Our understanding of the technology and uses of PBCs is documented above. However, a topic that has received far less attention is the role of wider systems and governance in determining whether PBCs become widely planted (Silveira & Johnson, 2016). Historical studies of rapid and profound transitions occurring in other industries such as energy (Fouquet & Pearson, 2012) and transport (Evans, 1981) have demonstrated that transitions are not necessarily led by experts or driven by technology, and are unlikely to be rationally planned or linear. The key feature of many of these studies is an appreciation of the socio‐technical regime; the idea that policy makers, technology users and scientists all participate in the co‐creation and development of a technology, rather than viewing technology and its uptake as a purely technical issue (De Laurentis, 2015).

Geels and Schot (2007) provide a useful (but critiqued) framework for transition using the multi‐level perspective. Three levels exist. The highest level is the socio‐technical landscape. This consists of over‐arching factors such as cultural norms, macro‐economics and political traditions. These are relatively slow to change and, at least in the short term, are not influenced by other levels. The (second) meso‐level is the socio‐technical regime: the interactions between science, policy, industry, market preferences, regulation, culture and technologies in current use. These meso‐level regimes are seen as relatively stable and ‘locked in’ to particular patterns and interactions. The third level is known as niches and is where innovations begin; small networks of innovators act to incubate specific innovations.

In order for a technological transition to become established (i.e. a breakthrough of an innovation from being niche to being part of the wider socio‐technical landscape, interactions between all three levels are needed. When applied to the general question of uptake of PBCs several key themes emerge. First, at the level of the regime, cooperation and development of understanding between several very different industries is required. Second, the lack of understanding among innovators about non‐technical aspects of the regime is significant. An obvious example is labelling PBCs as being suited to growing on ‘marginal land’ (which may be more sympathetically described as ‘less profitable’ or ‘problem’ land)). As discussed by Helliwell (2018) the label ‘marginal land’ fundamentally misunderstands farmer's values because farmers spend considerable effort improving their land, it is a source of pride to them, and therefore labelling land as marginal is dismissive and unhelpful because farmers always try to get to the best out of their land. Third, transition to an economy based on biomass is not being driven by the technology, rather it is the socio‐technical landscape (e.g. the need to limit climate change) and the regime (e.g. the reconfiguring of the energy industry towards renewable sources) that are driving the need for innovation. In addition, political events create new requirements that hasten change. For example in the oil industry vertical wells have been replaced by horizontal wells driven by a need to reduce costs (Pendleton, 1991). Pioneers of change respond to pressures from the socio‐technical landscape and regime, accept the need for co‐design of systems, and do not consider their work as being a purely scientific endeavour (De Laurentis, 2015; Roesler & Hassler, 2019).

Another key theme is the importance of understanding path dependency within the regime, and the extent to which it limits the potential for uptake of niche innovations. The availability of bio‐plastics for example, or the existence of strawboards suitable for use in the construction industry are not in themselves sufficient to overcome regime level factors such as economies of scale or lock‐in to existing infrastructures (Gottinger et al., 2020).

Consequently, when seen in the context of the multi‐level perspective, predicting how any transition will occur is fraught with difficulties. Historical transitions have been non‐linear, highly localised in their initial stages, and acutely influenced by where they can be tailored to reward. Current ‘landscape’ level changes such as rapid global warming and its public awareness, and the global energy crisis, have created a socio‐economic environment that supports change. In addition, biomass‐based energy and products can potentially contribute to 11 of the 17 UN Sustainable Development Goals (SDGs) (BMEL & BMBF, 2020). Therefore, we witness an impetus to create policies that support technology to displace fossil resources.

We agree with the six interacting policy approaches identified by Murphy‐Bokern (2016) (prioritising climate protection, market‐based interventions, standards, long‐term commitment and planning, research, land usage policies) as being required to support an expansion in PBC crop areas and usage in the EU and UK. These are all driven by the price of carbon and when combined have the potential to enable scaling up of PBC production. It should be noted that carbon pricing can be affected in many ways, such as a tax on carbon emission and/or embedded carbon or a credit for mitigating or storing carbon, both of which could be traded on the open market.

First, for prioritising climate protection, PBCs have the advantage of high output returns relative to input costs, therefore achieving high energy ratios and low embedded greenhouse gas emissions. GHG balance and mitigation assessments need to include soil carbon changes due to land‐use change as well as a comparison with the previous land use (and other opportunity costs). Second, market‐based interventions or incentives need to ensure adequate profit for all actors in the production and utilisation chains for thermal generation of heat and electricity or bio‐based products. For example, in the UK there have been a basket of these incentives for energy including Renewable Obligation Certificates (ROCs), feed‐in‐tariffs (FIT), renewable heat incentives (RHI), Renewable Energy Guarantees of Origin (REGO), Smart Export Guarantee (SEG) and contracts for difference (CfD). The CfD is the UK's new main mechanism for supporting low‐carbon electricity because it guarantees a price reflecting the investment and does not change with market forces over the agreed lifespan. CfD guarantees return on investment for the producer and protects the consumer from unplanned market pressures such as war. We believe that variants of CfD could also effectively support bio‐based products because they could be tailored to reward developers for production, conversion and circularity. Such approaches need to incorporate demand‐side innovations with labelling, procurement and standardisation. Third, standards for bio‐based products and circularity are seen as key enabling technologies; however, as biomass types are diverse, standards are difficult to define. For example, the standards for wood pellets https://enplus‐pellets.de/ (accessed 12 Sept 2022) cannot be successfully applied to other pelletised biomass coming from PBCs due to different chemical compositions. For international trade to develop in bio‐based products further standards need to be developed. Fourth, long‐term commitment and planning are crucial as already highlighted in the Delphi analysis above. This is due to lead in times of 3–8 years for planting of crops and for construction of bioconversion facilities which need to occur at the same time to avoid ‘chicken and egg’ stagnation (Flavell, pers. comm). Fifth, research policies are needed to accelerate PBC breeding and agronomy to reduce establishment times on available land types, improve resilience to drought, frost and heat, increase yields and improve biomass quality. Research is also needed to integrate top‐down GIS methods, informed by images from drones and satellites, with bottom‐up social science approaches to support land managers who are considering including PBCs in their business portfolios. Land managers need to be included in the development of measurement, reporting and verification systems aiming to quantify environmental, biodiversity and GHG mitigation benefits. This will aid the creation of a sustainable and validated carbon market supported by a credible life cycle assessment. Sixth, land‐use policies are needed to enable land managers to optimise resources and maximise profitability based on a combination of crop choice, available skill, on‐ and off‐farm infrastructure, personnel values and traditions. Careful analysis is needed to pitch the levels of payments required to stimulate planting PBCs and avoid triggering unintended consequences on food systems, soils or ecosystems. Environmental benefits may not be as simple as selecting the most challenging land for PBCs but it is better than a historical blanket 10% of CAP. New forms of farm payment, for example, UK Environmental Land Management Schemes (ELMS) recognise and reward environmental benefits in line with the principle of public money for public goods. Currently these schemes only make a small contribution to total farm income, but should provide a mechanism promote environmentally sound land‐use decisions.

All these policies have to march in step so that landowners, industrialists and their supporting scientists and policy makers join forces to translate PBCs into significant negative emission technologies to fight the climate emergency. This will require the provision of much‐needed information to the general public and an increase in the number of specialists throughout the PBC value chains, achieved through improved education at primary, secondary and tertiary levels, including apprenticeship schemes, with all contributing to ‘Shaping the Transition to a Sustainable, Biobased Economy’ (Lewandowski et al., 2018).

## 4 CONCLUSIONS

1. Land managers will ultimately determine how much land is allocated to PBCs for biomass production. Their decisions will be influenced by market demand for feedstock and confidence in the stability of the supply chain. They should be incentivised through specific policy measures coupled to carbon pricing. The percentage allocation of land to PBCs needs to be managed at a government level through incentives to avoid unintended consequences such as loss of biodiversity or reduction in essential food security.
2. Reward mechanisms are required for commercial developers of low‐carbon bioenergy and bio‐based products to encourage investment in a way that rewards actors in the entire value chain, particularly the farmers. This will require further development of measurement, reporting and verification systems to ensure that payments are made for actual long‐term GHG emission mitigation.
3. Support for innovation in the research and development (R&D) of biomass production to increase the availability of planting material to upscale to the hectarage required for net zero.
4. Support for utilisation, both energetic and material, with public–private collaborations should continue until higher technological readiness levels are achieved for the whole value chain including cascaded use of the feedstocks and products.
5. Long‐term research is needed to quantify the impact and value of large‐scale PBC introduction into the landscape on ecosystem functions including carbon sequestration to soil, carbon mitigation, flood prevention, erosion control, water cycling, water quality, soil fertility, biodiversity and cultural values. The value of these ecosystem benefits may be of the same order as the biomass value chain.
6. Interdisciplinary training and education is required to develop the body of expertise and experience for growing the PBC industry to improve the pool of skilled workers.
7. Funding for ‘on‐farm’ innovation for agronomy, harvest, transport and storage with comparative sustainability assessments.
8. Our policy recommendations are to:
  - support ramp up of PBC production from less than 1% to 10% of farmed land by 2050 by incentivising farmers in Europe;
  - involve the community during the process of project development;
  - secure long‐term commitments to public‐supported R&D between national and EU‐funded projects and coordinate between them;
  - to support industry involvement in projects for commercial translation of the technologies developed.

## CONFLICT OF INTEREST

The authors declare that progress reported in this paper, which includes input from industrial partners, is not biased by their business interests.

## Supporting information


Appendix S1:

## Data Availability

The data that support the findings of this study are openly available in dryad at https://doi.org/10.5061/dryad.w6m905qtf.
