# Supplementary material for: Perennial biomass cropping and use: Shaping the policy ecosystem in European countries
Source: Glob Change Biol Bioenergy. 2023 Mar 13;15(5):538–58. doi: 10.1111/gcbb.13038 (PMC10946487; doi:10.1111/gcbb.13038)
Supplement: Supplementary file 1 — Appendix S1: [file GCBB-15-538-s001.docx]

**Appendix**

Table S1. Details of projects that have supported the development of the PBC *Miscanthus* in UK and Europe, giving a breakdown of the topics covered and showing the R&D gaps.

Figure S1. Number of reported yields by stand age for papers published on *Miscanthus* as an example PBC from 1992 to 2022.
